# N-Glycosylation of the Na+-Taurocholate Cotransporting Polypeptide (NTCP) Determines Its Trafficking and Stability and Is Required for Hepatitis B Virus Infection

**DOI:** 10.1371/journal.pone.0170419

**Published:** 2017-01-26

**Authors:** Monique D. Appelman, Anindita Chakraborty, Ulrike Protzer, Jane A. McKeating, Stan F. J. van de Graaf

**Affiliations:** 1 Tytgat Institute for Liver and Intestinal Research, Academic Medical Center, Amsterdam, the Netherlands; 2 Institute of Virology, Technische Universität München / Helmholtz Zentrum München, München, Germany; 3 Institute for Advanced Study, Technische Universität München, München, Germany; 4 Centre for Human Virology, University of Birmingham, Birmingham, United Kingdom; 5 Department of Gastroenterology and Hepatology, Academic Medical Center, Amsterdam, the Netherlands; National Institute of Infectious Diseases, JAPAN

## Abstract

The sodium/bile acid cotransporter NTCP was recently identified as a receptor for hepatitis B virus (HBV). NTCP is glycosylated and the role of glycans in protein trafficking or viral receptor activity is not known. NTCP contains two N-linked glycosylation sites and asparagine amino acid residues N5 and N11 were mutated to a glutamine to generate NTCP with a single glycan (NTCP-N5Q or NTCP- N11Q) or no glycans (NTCP- N5,11Q). HepG2 cells expressing NTCP with a single glycan supported HBV infection at a comparable level to NTCP-WT. The physiological function of NTCP, the uptake of bile acids, was also not affected in cells expressing these single glycosylation variants, consistent with their trafficking to the plasma membrane. However, glycosylation-deficient NTCP (NTCP-N5,11Q) failed to support HBV infection, showed minimal cellular expression and was degraded in the lysosome. This affected the physiological bile acid transporter function of NTCP-N5,11Q in a similar fashion. In conclusion, N-glycosylation is required for efficient NTCP localization at the plasma membrane and subsequent HBV infection and these characteristics are preserved in NTCP carrying a single carbohydrate moiety.

## Introduction

The sodium-dependent taurocholate cotransporting polypeptide (NTCP) is an integral membrane glycoprotein that participates in the enterohepatic circulation of bile acids [1]. NTCP is expressed exclusively at the basolateral membrane of hepatocytes in the liver [2] and mediates the uptake of glycine/taurine-conjugated bile acids from the portal vein [3–5].

NTCP was recently identified as the bona fide receptor for hepatitis B virus (HBV) and hepatitis delta virus (HDV) [6]. HBV and HDV share the same envelope consisting of a membrane lipid bilayer into which the small (S), medium (M) and large (L) HBV envelope glycoproteins are integrated. All three envelop proteins share the S domain, but M and L N-terminally have an additional preS2 or preS1 and preS2 domain, respectively. L is responsible for the specific receptor binding via amino acids 157–165 in its pre-S1 domain, which interact with the third extracellular domain of NTCP [6]. Silencing NTCP expression in HepaRG cells inhibits HBV and HDV infection and exogenous expression in hepatocyte-derived cell lines HepG2 and Huh-7 confers permissivity to both viruses [6, 7].

HBV or HDV particle entry into hepatocytes occurs via a multistep process and NTCP binding is preceded by a low affinity attachment of the virus to cells via the carbohydrate side chain of heparin sulfate proteoglycans (HSPGs) [8]. Glypican 5 was identified as one of the HSPGs involved in this process [9]. We recently reported that lentiviral pseudotypes expressing HBV glycoproteins infect human hepatoma cells in an NTCP-dependent manner [10] supporting the model that NTCP envelop-protein interaction is sufficient to induce particle uptake and membrane fusion. Moreover, HBV infection of HepG2-NTCP cells is inhibited in a concentration-dependent manner by taurine and glycine conjugates of bile acids, the main substrates for NTCP [11–14], suggesting that bile acids and the HBV pre-S1 domain compete for NTCP binding. Indeed, a synthetic N-acylated pre-S1 lipopeptide that binds NTCP, Myrcludex B, inhibits HBV infection in vitro and in vivo [7, 11, 15–17]. Specific domains of human NTCP are essential for viral infection and exchange of the first extracellular loop of the human homolog (specifically amino acids 84–87) into the murine counterpart confers susceptibility to HBV/HDV infection [6]. However, detailed understanding on the structural requirements of viral docking to NTCP and the role of carbohydrate moieties in this process is lacking.

**Fig 1:**
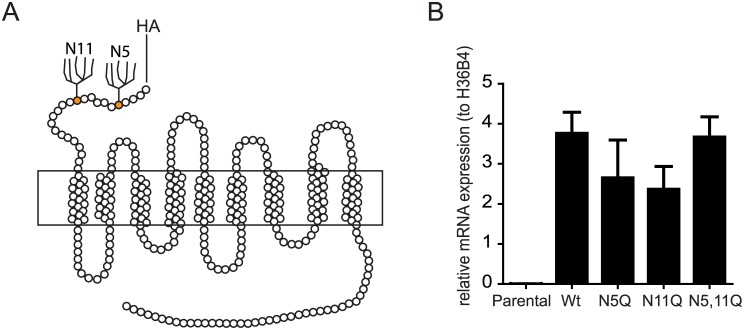
NTCP structure and mRNA expression. (A) Structure of human NTCP (hNTCP). An HA tag was present at the cell surface exposed N-terminus. N-linked glycosylation sites are indicated. (B) NTCP mRNA expression in HepG2 cells expressing NTCP-WT, NTCP-N5Q, NTCP-N11Q or NTCP-N5,11Q analysed by qRT-PCR using primers listed in S2 Table. NTCP expression values were calculated relative to housekeeping gene H36B4. Data represent mean +/- SD (N = 4).

Human NTCP is a multi-pass transmembrane protein that comprises an extracellular N-terminus and cytoplasmic C-terminal domain [5]. The extracellular N-terminal domain encodes two N-glycosylation sites at residues N5 and N11 (Fig 1A) [5, 18] and we hypothesize that these glycosyl moieties regulate NTCP trafficking and localization at the plasma membrane. Carbohydrate moieties of glycoproteins play several roles, such as controlling protein folding, stabilizing protein conformation, intracellular and membrane trafficking and interaction with other proteins [19]. We therefore investigated the role of N-glycosylation on NTCP receptor activity for HBV and bile acid transporter function.

## Results

### N-glycosyl moieties of NTCP are required for HBV infection

Site-directed mutagenesis of a tagged NTCP open reading frame introduced single or double substitution of the exofacial Asn residues (N5 and N11) with glutamine, resulted in four constructs; NTCP-wild type (WT), NTCP-N5Q, NTCP-N11Q and NTCP-N5,11Q. Expression of WT and NTCP mutants in HepG2 hepatoma cells showed comparable levels of mRNA as determined by qPCR (Fig 1A).

**Fig 2:**
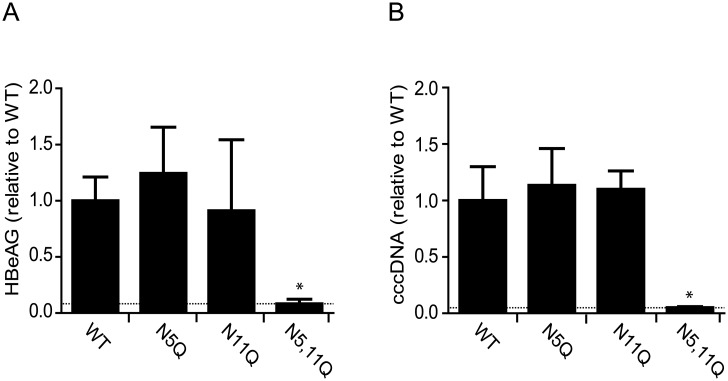
HBV infection of wild type and glycosylation deficient NTCP variants. HepG2 cells stably expressing NTCP-WT, NTCP-N5Q, NTCP-N11Q or NTCP-N5,11Q were infected with HBV. (A) 3 days post infection, HBeAg production was measured by ELISA. (B) Establishment of HBV infection was determined by quantification of intracellular cccDNA 3 days post-infection. Values are given as mean +/- sd of two independent experiments each performed in triplicate. The dotted line represents the values obtained after infecting parental HepG2 cells that lack NTCP expression. *Significantly different from NTCP-WT values (p < 0.05).

We first examined the effect of N-glycosylation on HBV infection of NTCP expressing HepG2 cell lines. HBV establishes a covalently closed circular DNA (cccDNA) in the nucleus of infected cells that serves as the transcription template for all viral proteins including secreted hepatitis B e antigen (HBeAg). Infection was assessed by quantifying cccDNA by qPCR and measuring HBeAg expression. HBV infection of HepG2 cells expressing WT or single glycan mutants, NTCP-N5Q and NTCP-N11Q, resulted in comparable levels of cccDNA and HBeAg expression (Fig 2). However, disruption of both glycosylation sites (NTCP-N5,11Q) prevented viral infection, both at a multiplicity of infection (MOI) of 100 (Fig 2), and 400 (data not shown). Since cells expressing NTCP with a single glycan located either at N5 or N11 support similar levels of infection as the WT transporter molecule, we conclude that a single glycan is required for HBV infection. Moreover, the location of this glycan is not critical since both N5 and N11 mutants support similar levels of viral infection.

### Bile acid uptake affected by NTCP glycosylation pattern

**Fig 3:**
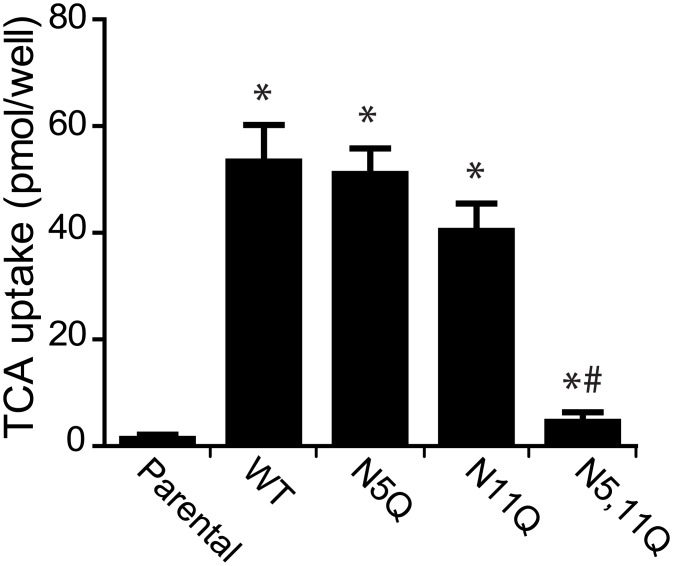
Effect of NTCP glycosylation on bile acid transport activity. Taurocholate (TCA) uptake assay in parental HepG2 cells and those stably expressing NTCP-WT, NTCP-N5Q, NTCP-N11Q or NTCP-N5,11Q. Cells were incubated for 2 minutes with uptake buffer containing 20 mM taurocholate, spiked with [^3^H]-taurocholate. NTCP specific uptake capacity is defined as pmol TCA uptake/well, where the bars represent the mean +/- sd of three experiments each performed in quadruplicate. *Significantly different from parental cells and # indicates significantly different from NTCP-WT values (p < 0.05).

Since HepG2 cells expressing the glycosylation deficient NTCP (NTCP-N5,11Q) variant failed to support HBV infection, we investigated the capacity of these cells to uptake bile acids. The single mutants, NTCP-N5Q and NTCP-N11Q, exhibited TCA transport activity comparable to NTCP-WT. However, NTCP-N5,11Q showed a minimal uptake (Fig 3), suggesting that NTCP lacking both N-glycosyl moieties is unable to transport bile acids. The reduced uptake capacity of NTCP-N5,11Q was also observed in Huh-7 cells (S1A Fig). Furthermore, the limited transport of bile acids by NTCP-N5,11Q was not due to the HA-tag since untagged NTCP-N5,11Q showed a similar phenotype (S1C–S1E Fig).

### NTCP N-glycosylation profile

**Fig 4:**
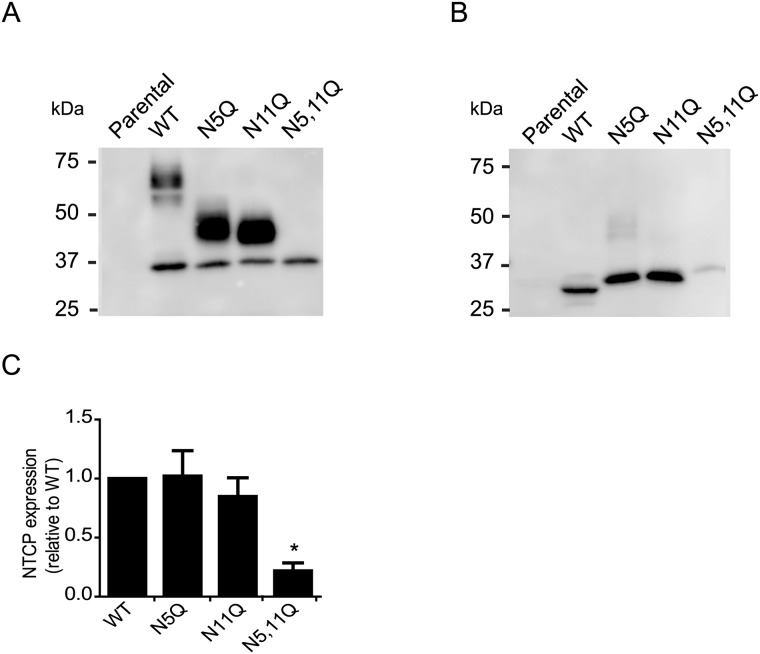
Effect of N-linked glycosylation on NTCP protein expression. (A and B) HA-tagged NTCP was immunoprecipitated (IP) from lysates of HepG2 cells stably expressing NTCP-WT, NTCP-N5Q, NTCP-N11Q or NTCP-N5,11Q. IP samples were subjected to immunoblotting for NTCP (using anti-HA-hrp). (B) IP samples were digested with PNGase F for 1h at 37°C (500 units) prior to immunoblotting for NTCP. (C) NTCP was quantified by image J and expressed relative to NTCP-WT. Molecular mass is given in kDa on the left-hand side. Results are mean +/- sd. *Indicates significant different from NTCP-WT (p <0.05).

Since HBV infection and bile acid uptake was defective in HepG2 cells expressing NTCP-N5,11Q, we investigated the effect of this mutation on protein expression. NTCP-WT was detected as multiple bands with a molecular mass of 35-60kDa. HepG2 cells expressing NTCP carrying a single glycan moiety (N5Q or N11Q), displayed a band of 45kDa and NTCP-N5,11Q migrated around 35kDa (Fig 4A). To test whether the bands at 35-60kDa correspond to differences in N-glycosylation, samples were treated with PNGaseF, that cleaves N-glycan chains from glycoproteins. This procedure resulted in a near complete shift of NTCP signal towards +/- 35kDa (Fig 4B), demonstrating that the differences in molecular weight were due to the addition of N linked glycans on the protein. Since comparable protein amounts were loaded we conclude that the glycosylation-depleted NTCP variant shows a modest level of protein expression (Fig 4C). Furthermore, similar glycosylation patterns were observed in HepaRG and Huh7 cells (S1B Fig).

### Glycosylation is essential for NTCP localization at the plasma membrane

**Fig 5:**
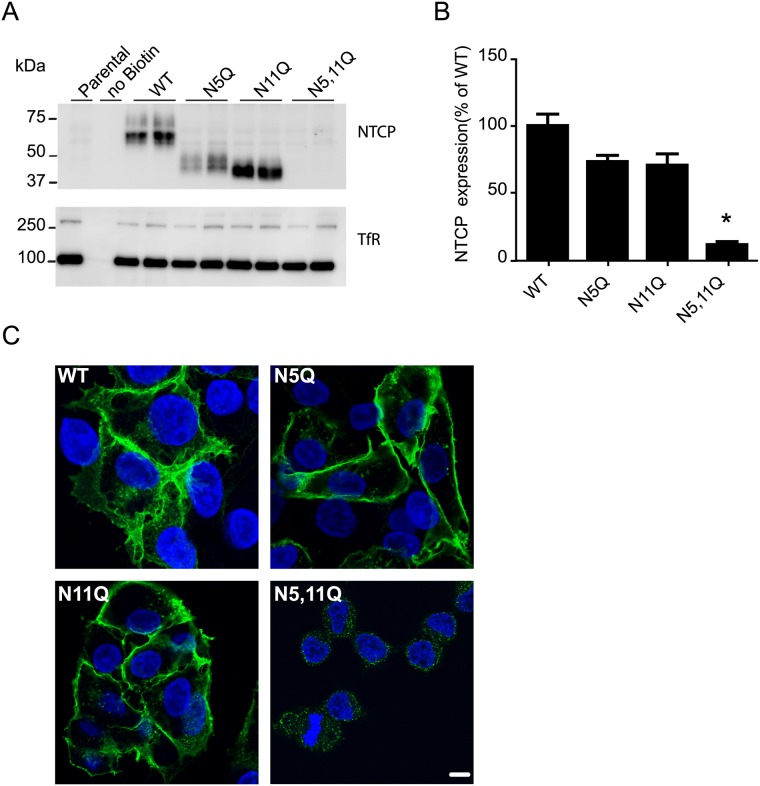
Effect of N-linked glycosylation on NTCP plasma membrane expression. Plasma membrane expression was assessed by surface-biotinylation of parental HepG2 cells or those expressing NTCP-WT, NTCP-N5Q, NTCP-N11Q, NTCP-N5,11Q. (A) Representative immunoblot for NTCP membrane expression and for the Transferrin receptor (TfR), as a loading control. Molecular mass is given in kDa on the left-hand side. (B) NTCP expression at the plasma membrane was semi-quantified by Myrcludex-B-FITC intensity. Fluorescence of the parental cells was subtracted before normalization and the net fluorescence units expressed relative to NTCP-WT. *Indicates significant different from NTCP-WT (p<0,05) (C) Confocal microscopic images of HepG2 cells expressing NTCP-WT or mutant proteins stained with anti-HA (green) and counterstained with Hoechst (blue). Scale bar represents 10μm.

Since HBV infection and bile acid uptake requires NTCP localization at the plasma membrane, we anticipated that N-glycosylation would influence NTCP expression at the plasma membrane. Cell surface biotinylation demonstrates that NTCP-WT, NTCP-N5Q, NTCP-N11Q but not NTCP-N5,11Q were detected in the plasma membrane fraction (Fig 5A). Notably, the minority pool of unglycosylated WT protein, migrating at ~35 kDa (Fig 4A) is also not expressed at the cell surface (Fig 5A). Transferrin receptor was used as loading control and similar expression levels were detected in all samples. These results were similar when NTCP cell surface abundance was measured with FITC-labeled Myrcludex B. Myrcludex B, a ligand based on the pre-S1 domain of HBV-L protein [7, 11, 15], binds NTCP at position 157–165, so at a certain distance from the glycosyl moieties. NTCP with a single glycan moiety showed a modest reduction in plasma membrane expression compared to NTCP-WT (Fig 5B). However, NTCP-N5,11Q showed a significant reduction in plasma membrane expression compared to NTCP-WT (Fig 5B). Altogether, this suggests that glycosylation-depleted NTCP, in contrast with NTCP-N5Q and NTCP-N11Q, is not efficiently expressed or targeted to the plasma membrane.

Since NTCP-N5,11Q showed only minimal plasma membrane expression, we tested whether this protein was retained in the cytoplasm. As shown in Fig 5C, HepG2 cells expressing NTCP-N5,11Q displayed a weak intracellular signal. The NTCP mutants with one glycan (NTCP-N5Q, NTCP-N11Q), in contrast, showed the same NTCP distribution as NTCP-WT.

### Glycosylation-depleted NTCP is rapidly degraded in lysosomes

**Fig 6:**
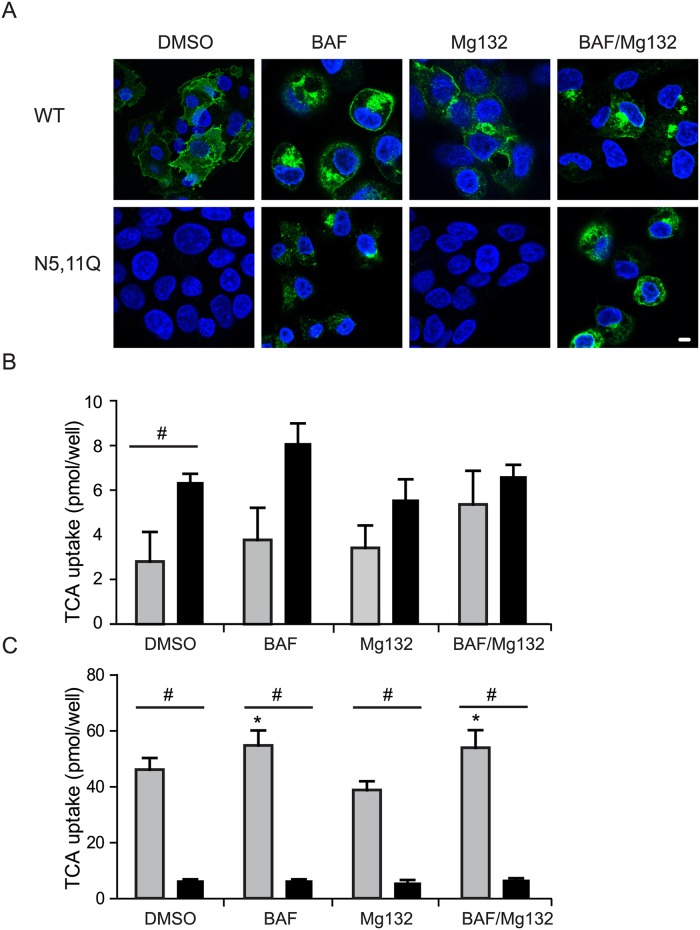
Treatment with Bafilomycin but not with MG132 induces expression of NTCP-N5,11Q. (A,B) Parental HepG2 or cells expressing NTCP-WT or NTCP-N5,11Q were treated with 0.1% DMSO, Bafilomycin (10nM/24h), MG132 (20μM/6h) or a combination of Bafilomycin (10nM/24h) and MG132 (20μM/ for the last 6h). (A) Immunofluorescence microscopy of NTCP-WT and NTCP-N5,11Q. in which NTCP was visualized using anti-HA antibody (green) and Hoechst (blue) was used to visualize the nucleus. (B) Results of TCA uptake in parental HepG2 cells (grey) and HepG2 cells expressing NTCP-N5,11Q (black). (C) Results of TCA uptake in HepG2 cells expressing NTCP-WT (grey) or NTCP-N5,11Q (black). (B,C)The uptake capacity of the NTCP was determined as pmol TCA uptake/well. Bars represent the mean +/- sd of three independent experiments, each performed in quadruplicate. *Indicates significant different from DMSO treated (p <0.05). # indicates significant different between the cell-lines in the same treatment (p<0.05).

The low expression levels of NTCP N5,11Q may reflect an enhanced degradation of this variant. To investigate whether NTCP-N5,11Q was targeted to the proteasome or alternatively degraded in the lysosome, parental HepG2 cells and those stably expressing NTCP-WT or NTCP-N5,11Q. were treated with MG132, Bafilomycin or the combination of these two drugs. The proteasome inhibitor MG132 had no effect on NTCP-N5,11Q expression at the plasma membrane (Fig 6A) or bile acid uptake (Fig 6B and 6C). However, treatment with Bafilomycin to prevent degradation of internalized and lysosomally targeted proteins [20], induced an intracellular accumulation of NTCP (Fig 6A). The increased intracellular signal of NTCP-N5,11Q following Bafilomycin treatment either alone or in combination with MG132 was not accompanied by restoration of bile acid uptake activity (Fig 6C). Activity of NTCP-WT was not affected by Bafilomycin or MG132 (Fig 6C). In conclusion, NTCP-N5,11Q is rapidly endocytosed to be degraded in lysosomes.

## Discussion

In the present study, we show that N-linked glycans on NTCP are required for protein expression, localization, bile acid uptake and NTCP-mediated HBV infection. NTCP binds the preS1 domain of the HBV encoded L protein with high-affinity, and its selective expression at the basolateral hepatocellular plasma membrane can explain both liver tropism and species specificity of HBV and HDV.

This study aimed to assess whether the glycosyl-moieties of NTCP itself contribute to virus infection. Strategies to modulate total cellular glycosylation, such as using tunicamycin would not be suitable to tackle specifically the contribution of NTCP-bound glycans to HBV infection. In NTCP, both N5 and N11 are used as glycan-attachments residues and mutating both amino acids abolished NTCP glycosylation. These amino acids are conserved across multiple species, including human, mouse, rat, macaca, pongo, and cow and in the related transporter Slc10a2/ASBT (S3 Table). The data presented here demonstrate that at least one glycan residue is sufficient for NTCP, plasma membrane localization, bile acid uptake and viral receptor activity. NTCP-N5,11Q is degraded via the lysosome. This could imply that the glycosylation-deficient NTCP might be first targeted to the plasma membrane, to be rapidly internalized and degraded. This model is supported by data showing a low level of bile acid uptake in HepG2 cells expressing NTCP-N5,11Q compared to parental cells. However, treatment with Bafolimycin to inhibit lysosomal degradation did not enhance plasma membrane abundance of the glycosylation-deficient NTCP, nor bile acid uptake. For several other integral membrane proteins, N-glycosylation is associated with decreased protein stability and absence of glycosyl moieties can promote degradative pathways [21–23]. Interestingly, multiple transporters that lack their normal glycosylation, degradation occurs via the proteosomal route, in line with ER-assisted degradation and impaired trafficking to the plasma membrane [23, 24]. For NTCP the degradation occurs via a lysosomal fashion, suggesting a post-ER sensing of altered NTCP conformation and degradation after entering the endosomal pathway. The small but significant bile acid uptake seen for NTCP-N5,11Q is in line with (partial) plasma membrane targeting. The significant uptake of taurocholate that can be detected even while plasma membrane abundance is minimal also implicates that the NTCP N5,11Q protein is functional. This is similar to some other glycoproteins with a transporter function, for example ASCT2, where only membrane trafficking and not the transport function is affected when glycosylation sites are mutated [22]. In contrast, for other transporters, for example PEPT1 N-linked glycosylation does not affect plasma membrane abundance in Xenopus laevis oocytes, but transport kinetics are lower in the unglycosylated mutant [25].

We did not observe any evidence of HBV infection of HepG2 cells expressing NTCP-N5,11Q. This could suggest that i) viral entry requires a certain NTCP density at the cell surface, ii) NTCP needs to remain at the plasma membrane sufficiently long to allow efficient virus docking, or iii) the lysosomal degradation of NTCP is not compatible with its role in the viral life cycle. Since NTCP-N5,11Q was not detected at the plasma membrane by cell surface biotinylation, we conclude that glycosylation plays an important role in NTCP trafficking. This phenotype makes it impossible to reliably assess the contribution of the NTCP glycan moieties on viral binding per se to the receptor. However, we can conclude that one glycan residue is sufficient for bile acid uptake as well as efficient HBV entry. Furthermore, we established that glycosylation of NTCP, at least one glycan residue, is required for NTCP localization at the plasma membrane and subsequent bile acid uptake.

## Methods

### Chemicals and reagents

Hygromycin (merckmillipore), anti-HA HRP (sigma), ^3H^Taurocholate (Perkin Elmer), Taurocholate (Sigma), MG132 (Z-Leu-Leu-Leu-al) (sigma), Bafilomycin A1 (sigma), DMSO (VWR), Myrcludex B-FITC (Pepscan), Transferrin receptor (Invitrogen), anti-mouse-HRP(DAKO).

### NTCP glycosylation mutants

Aspargine to glutamine mutations in NTCP constructs [26] were generated using the QuikChange^®^ Site-Directed Mutagenesis Kit (Agilent Technologies). NTCP open reading frames were cloned into the vector pLenti-PGK-Hygro-DEST (Addgene) using the Gateway LR clonase II enzyme mix (Invitrogen) after initial cloning into pENTR-D-TOPO according to the manufacturer’s instructions (Life Technologies). All constructs were sequenced to verify they contained the correct mutation. Primers used to generate the constructs can be found in S1 Table.

### Cell culture

Human hepatocellular carcinoma cells (HepG2, from ATCC, VA, USA) and human embryonic kidney (HEK293T, from ATCC) cells were cultured in Dulbecco's modified Eagle's medium (Sigma) supplemented with 10% fetal bovine serum, 1% penicillin/streptomycin and, 1% Glutamine. Cells were grown at 37°C in a humidified incubator at a 5% CO_2_ atmosphere.

### Stable cell-line

HEK293T cells were seeded in 10cm^2^ plates, 24h before transfection with 3^rd^ generation virus plasmids, PMD2G, PMDL and PRSV vectors and one of the NTCP constructs. Medium of the HEK293T cells was harvested and added to HepG2 cells for 6 hours followed by refreshing of the medium. After 48h, the infected HepG2 cells were selected using Hygromycin (50 μg/ml).

### Determination of HBV infection

HepG2 cells were seeded onto collagen-coated plates (6x10^5^ cells/well) and after 3 days of differentiation using 2.5% DMSO infected with HBV (MOI = 100) overnight. Infection was proven by establishment of nuclear cccDNA and HBeAg secretion. HBeAg was detected in the culture medium using a commercial immunoassay (Siemens Molecular Diagnostics, Marburg, Germany). Total DNA was extracted from infected cells 3 days post infection (PI) using a NucleoSpin tissue kit (Macherey Nagel, Düren, Germany) and subsequently treated with T5 exonuclease (10U/μl) for 10 min before cccDNA detection. HBV cccDNA was detected by real time PCR (qPCR) with selective PCR primers as previously described [27, 28]. qPCR was performed on the LightCycler 480 real time PCR 96-well system II (Roche, 5015278001) and analysed using the second derivative maximum approach that takes normalisation to a reference gene (PrP) and primer efficiency into account.

### Immunofluorescence NTCP

HepG2 cells were seeded onto coverslip and cultured using standard culturing conditions until 60–70% confluence. 72h after seeding, cells were fixed with 3.7% formaldehyde/PBS for 20 min and permeabilized for 2 min with 0.2% Triton X-100/PBS at room temperature. After rinsing with PBS, nonspecific binding of antibodies was blocked by 2%(w/v) bovine serum albumin in PBS for 30 minutes at room temperature. Cells were incubated with mouse anti-HA (H9658, Sigma) for 2 hours followed by Alexa Fluor^™^ 488-conjugated goat anti-mouse IgG (Invitrogen) for 45 minutes. Cover slips were washed three times with PBS and Hoechst staining was performed afterwards. Cells were mounted with mowiol. Images were obtained using a confocal laser scanning microscope (TCA SP8 X LEICA) equipped with HC Plan Apochromat 63X NA 1,4 oil CS2.

### Taurocholate uptake assay

HepG2 cells were seeded into 24-well plates and cultured using standard culturing conditions until they reached 80% confluence. Cells were rinsed twice with warm uptake buffer [5 mM KCl, 1.1 mM KH_2_PO_4_, 1 mM MgCl_2_, 1.8 mM CaCl_2_, 10 mM D-glucose, 10 mM Hepes and 136 mM NaCl]. After which, cells were incubated at 37°C with uptake buffer containing 20μM taurocholate supplemented with 0,25μC [^3^H]taurocholate for 2 min. Subsequently, cells were washed four times with ice-cold PBS and lysed in 0.05% SDS. Accumulation of radiolabelled substrates was determined by scintillation counting.

### Immunoblotting

Proteins were separated on a 10% polyacrylamide gel and transferred to nitrocellulose membrane. Unspecific binding was blocked with 5% milk in 0.1% TBS-TWEEN (TBST) for 1h at room temperature. Antibodies for either NTCP (visualized with HA-HRP) or transferrin receptor (TfR) were added to 5% milk-TBST and incubated with the membrane for 16h at 4°C while rocking. After three washes with TBST, membranes were incubated with a HRP (horseradish peroxidase)-conjugated mouse antibodies (Sigma) to visualize the TfR as described previously [26]. Following three washes in TBST, ECL was performed, and proteins were detected by chemiluminescence.

### Immunoprecipitation

Cells were grown in a 100mm culture plates to 80% confluence. After washing with PBS, cells were lysed in lysis buffer (150 mM NaCl, 50 mM Tris PH 7,4, 5 mM EDTA, 10% (w/v) sucrose, 1% Nonidet P40) supplemented with protein inhibitors. Subsequently, BCA assay according to manufactory protocol was performed and equal protein amounts were incubated with monoclonal anti-HA antibody (Sigma 9568) immobilized on Protein A—agarose beads (Sigma) for 16h at 4°C. Samples were analyzed by immunoblotting using anti-HA antibodies (Sigma) and HRP-conjugated mouse antibodies (Sigma) as described above.

### PNGaseF treatment

NTCP was immunoprecipitated from stably transduced HepG2 cells using monoclonal anti-HA antibody immobilized on Protein A-agarose beads as described above. After addition of sample buffer (2% (w/v) SDS, 10% v/v glycerol 40mM Tris pH6.8, Bromophenol blue (Sigma Alrich) containing 0.1M DTT (Promega), supernatant was taken and samples were treated with PNGase F (peptide *N*-glycosidase F) according to the manufacturer's instructions (New England Biolabs).

### Cell surface biotinylation assay

Cells were grown in a 100mm culture plates to 80% confluence. After washing with PBS-CM (PBS containing 1mM MgCl_2_, 0.5mM CaCl_2_, pH 8.0), 0,5mg/ml NHS-ss-Biotin/PBS-CM was added for 30min at 4°C. After quenching with PBS-CM containing 0.1% BSA, cells were washed three times with PBS-CM and lysed in lysis buffer (150 mM NaCl, 50 mM Tris PH 7,4, 5 mM EDTA, 10% (w/v) sucrose, 1% Nonidet P40) supplemented with protein inhibitors. Lysate was centrifuged and supernatant was added to pre-washed neutravidin beads. Pull down was performed for 2 hours followed by washing with lysis buffer. The proteins were eluted in sample buffer and subjected to immunoblotting as described above.

### Lysosomal versus proteosomal degradation

Cells were plated for either TCA assay or immunofluorescence as described above. 48 or 66 hours after seeding, treatment with either DMSO, 10nM Bafilomycin (24h) or 20μM MG132 (6h) or a combination of the last two was performed. After treatment, cells were analyzed following the above described protocols.

### NTCP plasma membrane expression

Cells were seeded into 96 wells plate (Corning; 734–1609) three days before staining. Cells were stained with 0.02μM Myrcludex-B—FITC for 30min at 37°C. Myrcludex B-Fitc intensity was measured using a NOVOstar microplate reader (BMG Labtech GmbH, Offenburg) at λex / λem = 483–14 nm/ 530–30 nm. The option orbital averaging was set at a diameter of 3mm. Background fluorescence was subtracted and results were plotted as percentage of NTCP WT.

### mRNA expression of NTCP

Cells were seeded in a 6 wells format, three days before RNA extraction. Total RNA was extracted with TRIzol reagent (Invitrogen, Bleiswijk, The Netherlands). Complementary DNA was synthesized from DNAse-treated total RNA with Oligo-dT_12-18_ and Superscript II reverse transcriptase (Invitrogen). Quantitative real-time polymerase chain reaction (qRT-PCR) was carried out in a Roche Lightcycler 480 II instrument using SYBR green (Roche) and the hNTCP primers listed in S2 Table. Expression level in each sample was normalized to reference gene H36B4.

### Statistical analysis

Values are expressed as mean ± sd. Significant analysis was performed using one-way analysis of variances following the Bonferroni’s multiple comparison test. Graphpad Prism software was used for analysis.

## Supporting Information

S1 Fig NTCP expression is similar in different cell-lines and not its function is not influenced by the HA-tag. (A) Taurocholate (TCA) uptake assay in parental Huh-7 cells and those stably expressing NTCP-WT, NTCP-N11Q or NTCP-N5,11Q. Cells were incubated for 2 minutes with uptake buffer containing 20 mM taurocholate, spiked with [^3^H]-taurocholate. NTCP specific uptake capacity is defined as pmol TCA uptake/well, where the bars represent the mean +/- sd (n = 4). (B). HepG2 cells, HepaRG cells and Huh-7 cells stably expressing NTCP-WT were lysed and equal amount of total protein were subjected to immunoblotting for NTCP either directly or after digestion with PNGase F for 1h at 37°C (500 units). (C) Taurocholate (TCA) uptake assay in parental HepG2 cells and those stably expressing NTCP-WT or NTCP-N5,11Q with or without a HA-tag. Cells were incubated for 2 minutes with uptake buffer containing 20 mM taurocholate, spiked with [^3^H]-taurocholate. NTCP specific uptake capacity is defined as pmol TCA uptake/well, where the bars represent the mean +/- sd (n = 4). (D,E) NTCP mRNA expression (D) and HA-NTCP mRNA expression (E) in HepG2 cells expressing NTCP-WT or NTCP-N5,11Q with or without a HA-tag analysed by qRT-PCR using primers listed in S2 Table. NTCP expression values were calculated relative to housekeeping gene H36B4. Data represent mean +/- SD (N = 4).
(EPS)

S1 Supplementary Methods HUH-7 cell culture conditions. (DOC)

S1 Table Primer sequences used for site-directed mutagenesis to generate single or double substitution of the two Asn residues (N5 and N11) with glutamine in HA-NTCP by site-directed mutagenesis. (DOC)

S2 Table Oligonucleotide primers used for qRT-PCR to analyze NTCP expression and the reference gene H36B4. (DOC)

S3 Table Overview of the conserved glycosylation sites in SLC10A1 across multiple species, including human, mouse, rat, macaca, pongo, and cow and in the related transporter Slc10A2/ASBT. (DOC)
