# Supplementary material for: N-Glycosylation of the Na+-Taurocholate Cotransporting Polypeptide (NTCP) Determines Its Trafficking and Stability and Is Required for Hepatitis B Virus Infection
Source: PLoS One. 2017 Jan 26;12(1):e0170419. doi: 10.1371/journal.pone.0170419 (PMC5268470; doi:10.1371/journal.pone.0170419)
Supplement: S1 Supplementary Methods — (DOC) [file pone.0170419.s002.doc]

**Supplementary Methods**

**Cell culture** *-* Hepatoma (Huh-7) cells were cultured in Dulbecco's modified Eagle's medium (Sigma) supplemented with 10% fetal bovine serum, 1% penicillin/streptomycin and 1% Glutamine. HepaRG cells were cultured in William’s E medium, 10 % FBS Fetalclone II (from Hyclone; both Thermo Scientific), 1% penicillin/streptomycin, 1% Glutamine, 0,023 IE/mL human insulin, 4,7 µg/mL hydrocortisone and Gentamicin. For differentiation, 1.8% DMSO were added when cells reach confluency and cells were further cultivated for 4 weeks. All cells were grown at 37°C in a humidified incubator at a 5% CO_2_ atmosphere. Stable cell-line was generated similarly as in HepG2 cells.
