# Supplementary material for: N-Glycosylation of the Na+-Taurocholate Cotransporting Polypeptide (NTCP) Determines Its Trafficking and Stability and Is Required for Hepatitis B Virus Infection
Source: PLoS One. 2017 Jan 26;12(1):e0170419. doi: 10.1371/journal.pone.0170419 (PMC5268470; doi:10.1371/journal.pone.0170419)
Supplement: S1 Table — (DOC) [file pone.0170419.s003.doc]

**Supplementary Table 1**

**S1 Table. Primer sequences used for site-directed mutagenesis to generate single or double substitution of the two Asn residues (N5 and N11) with glutamine in HA-NTCP by site-directed mutagenesis**.

| Mutation | Sense and antisense |
| --- | --- |
| hNTCP-N5Q | TCCATGGAGGCCCACCAAGCGTCTGCCCCATTC  GAATGGGGCAGACGCTTGGTGGGCCTCCATGGA |
| hNTCP-N11Q | GCGTCTGCCCCATTCCAATTCACCCTGCCACCC  GGGTGGCAGGGTGAATTGGAATGGGGCAGACGC |
