# Supplementary material for: N-Glycosylation of the Na+-Taurocholate Cotransporting Polypeptide (NTCP) Determines Its Trafficking and Stability and Is Required for Hepatitis B Virus Infection
Source: PLoS One. 2017 Jan 26;12(1):e0170419. doi: 10.1371/journal.pone.0170419 (PMC5268470; doi:10.1371/journal.pone.0170419)
Supplement: S2 Table — (DOC) [file pone.0170419.s004.doc]

**Supplementary Table 2**

**S2 Table. Oligonucleotide primers used for qRT-PCR to analyze NTCP expression and the reference gene H36B4.**

| Gene | Sense and antisense |
| --- | --- |
| NTCP | GGACATGAACCTCAGCATTGTG GCCGTTTGGATTTGAGGACG |
| H36B4 | TCATCAACGGTACAAACGA  GCCTTGACCTTTTCAGCAAG |
| HA-tag (NTCP) | CACCATGTACCCATACGATGTT  ATGATGCCATACTGTGCCAC |
