# Supplementary material for: N-Glycosylation of the Na+-Taurocholate Cotransporting Polypeptide (NTCP) Determines Its Trafficking and Stability and Is Required for Hepatitis B Virus Infection
Source: PLoS One. 2017 Jan 26;12(1):e0170419. doi: 10.1371/journal.pone.0170419 (PMC5268470; doi:10.1371/journal.pone.0170419)
Supplement: S3 Table — (DOC) [file pone.0170419.s005.doc]

**Supplementary Table 3**

**S3 Table**. **Overview of the conserved glycosylation sites in SLC10A1 across multiple species, including human, mouse, rat, macaca, pongo, and cow and in the related transporter Slc10A2/ASBT.**

| Species | Gene | Amino acid sequence | | | | | | | | | | | | | | |
| --- | --- | --- | --- | --- | --- | --- | --- | --- | --- | --- | --- | --- | --- | --- | --- | --- |
| Cow | SLC10A1 | M | E | A | F | N | E | S | S | P | F | N | F | S | L | P |
| Human | SLC10A1 | M | E | A | H | N | A | S | A | P | F | N | F | T | L | P |
| Pongo | SLC10A1 | M | E | A | H | N | A | S | A | P | F | N | F | T | L | P |
| Macara | SLC10A1 | M | E | A | H | N | A | S | A | P | F | N | F | T | L | P |
| Mouse | SLC10A1 | M | E | A | H | N | V | S | A | P | F | N | F | S | L | P |
| Rat | SLC10A1 | M | E | V | H | N | V | S | A | P | F | N | F | S | L | P |
| Human | SLC10A2 | M | N | D | P | N | S | C | V | D | N | A | T | V | C | S |
